# A postmortem grading system for articular cartilage loss of the dorsoproximal aspect of the proximal phalanx of Thoroughbred racehorses

**DOI:** 10.1177/10406387261467739

**Published:** 2026-07-29

**Authors:** Michaela J. Gibson, Keren E. Dittmer, Kylie A. Legg, Alvaro S. Wehrle-Martinez, Chris P. Beggan, Greg O. Sommerville, Chris W. Rogers

**Affiliations:** School of Veterinary Sciences, Massey University, Palmerston North, New Zealand; School of Veterinary Sciences, Massey University, Palmerston North, New Zealand; School of Veterinary Sciences, Massey University, Palmerston North, New Zealand; School of Veterinary Sciences, Massey University, Palmerston North, New Zealand; Waikato Equine Veterinary Centre, Cambridge, New Zealand; Cambridge Equine Hospital, Cambridge, New Zealand; School of Veterinary Sciences, Massey University, Palmerston North, New Zealand

**Keywords:** metacarpophalangeal joint, musculoskeletal, osteochondral chip fracture, racehorse, Thoroughbred

## Abstract

The metacarpophalangeal and metatarsophalangeal joints in Thoroughbred racehorses are common injury sites, given their high range of motion and compression during galloping. In New Zealand, the proximal phalanx (P1) is a common site for race-day fracture, but the lesions of this bone have not been described. Therefore, we created a scoring system to describe cartilage on the dorsoproximal aspect of P1 in New Zealand Thoroughbred racehorses. We collected 102 P1s from racehorses submitted for postmortem examination. Horses were categorized into 2 cohorts: 1) fracture (18 horses euthanized because of catastrophic fracture) or 2) control (16 racehorses euthanized for reasons unrelated to musculoskeletal injury). We used a modified 3-point scoring system to grade the dorsoproximal surface of P1 (from no damage [0] to severe damage [3]). The articular cartilage score did not differ between fracture and control cohorts (*p* > 0.05). Cartilage scores were higher in the medial aspect of the left forelimb and right hindlimb (*p* < 0.05), as expected given that those limbs experience greater strain when traveling in the counterclockwise race direction common in New Zealand. The articular cartilage score increased with increasing horse age and career starts (*p* < 0.05). Our results reflect the greater load on the medial aspect of the limb and reduced capacity of articular cartilage to repair as horses age.

The metacarpophalangeal (**MCPJ**) and metatarsophalangeal (**MTPJ**) joints have a high range of motion and are subjected to high compressive loads, particularly at the gallop. Consequently, the MCPJ is a common site for injury leading to lameness and lost training days in Thoroughbred racehorses.^18,30^ The components of the MCPJ—namely the metacarpal (**MC3**) condyles, sesamoid bones, and proximal phalanx (**P1**)—are the most common locations for race-day fracture, leading to early retirement or, in severe cases, euthanasia.^11,28^

Within the MCPJ, lesions on the palmar aspect of the articulating surfaces of the distal MC3 condyles have been the focus of much attention. This site is subjected to significant compressive forces because of the action of the MCPJ joint during maximal loading at midstance and propulsion.^12^ The compressive forces generate focal areas of high strain, with a localized sclerotic bone response or focal damage to subchondral bone.^7^ Clinically, these horses can be lame and have focal areas of radionucleotide uptake observed when examined with scintigraphy or positron electron tomography.^24,31^ Upon postmortem examination, localized partial or complete erosion of the articular cartilage can be observed. This collection of lesions is described as palmar osteochondral disease (**POD**) and a grading system and descriptive epidemiology of POD has been presented.^2,22,26^ Postmortem studies have estimated that 67–80% of Thoroughbred racehorses in the UK had POD in at least one condyle of the metacarpus or metatarsus, of which 14% had a POD lesion that was considered “severe.”^2,22^ Imaging studies have reported 72 of 131 (55%) horses screened for localized lameness had POD.^23^ Although POD may be associated with lameness, it does not appear to be associated with increased risk of fracture^8,27^ and may, in some situations, be considered protective for condylar fracture.^3,14^

The proximal sesamoid bones (**PSBs**) have also been the focus of much attention, given that they are frequently involved in fatal fractures within the distal limb and failure of the suspensory apparatus.^9^ However, descriptions of the lesions of the articulating surfaces of the PSBs are limited. Research on the PSBs tends to focus on describing changes in the suspensory ligament and the structural architecture and density of the PSBs.^3^

P1 is a common site of fatal race-day or training fracture, accounting for 16–17% of fatal limb fractures reported in flat racing.^11,20^ With increasing speed, the loading on the proximal surface of P1 increases and becomes highly focused on the dorsoproximal aspect and the sagittal groove.^4,5,19^ These sites of increased pressure on the articulating surface reflect the location of osteochondral bone chips, which typically are observed on the proximomedial aspect but can also be found on the proximolateral aspect.^32^ The occurrence of osteochondral bone chips increases in frequency with increasing horse age.^25^

Fatal fractures involving P1 are predominantly sagittal fractures, with the sagittal groove as point of origin. However, debate exists as to whether the dorsal or proximal aspect of P1 is the point of fracture origin; finite element analysis indicates that both have highly focal points of loading during stance.^19^ This focal loading generates changes in the bone architecture and would be expected to generate pathologic changes within the articulating cartilage at both sites. During an arthroscopic survey of 242 racehorses in the UK, wear lines were present on the proximal P1 articular cartilage in 81% of cases.^32^ The frequency of other lesions was much less, with full-thickness cartilage defect or erosion of dorsal P1 observed in only 12% of all cases.

Differences in the prevalence and presentation of MCPJ injuries and fatal fractures are well established between jurisdictions.^13,27^ These differences reflect the pattern of training load and the surfaces used for training and racing. The expectation is that these differences will be reflected in the lesions of the proximal P1 because the contact areas are greatly influenced by speed and track surface.^4^ However, without a methodology to quantify the articular cartilage lesions of P1, it is difficult to correlate these changes with clinical history.

Given differences in jurisdictions between training and racing patterns, the presentation and severity of lesions are likely to differ. Therefore, we aimed to create a scoring system to describe cartilage on the dorsoproximal aspect of P1 in New Zealand Thoroughbred racehorses affected and unaffected by forelimb fractures.

## Materials and methods

Whole distal forelimbs and hindlimbs were opportunistically collected from 34 Thoroughbred horses submitted for postmortem examination that had recently (<60 d) been in race training before euthanasia. Only horses that were participating in, or training for, flat races were included. Horses were categorized into 2 cohorts: 1) fracture (18 horses euthanized because of a catastrophic fracture) or 2) control (racehorses euthanized for reasons unrelated to musculoskeletal disease). We collected 102 first phalanges from these horses. All 4 P1s were collected from 15 horses, 3 P1s from 10 horses, 2 P1s from 3 horses, and 1 P1 from 6 horses.

### Fracture cohort

The fracture cohort consisted of distal limbs collected from Thoroughbred racehorses that had suffered a catastrophic fracture in a forelimb (**Table 1**)—either during race training, trials, or on race day—and were submitted for postmortem examination as part of the New Zealand Thoroughbred Racing–Massey University racehorse postmortem collaboration. As part of this collaboration, whole horses within a 3-h drive of Massey University are submitted to the School of Veterinary Science for postmortem examination. Given transport logistics, only the distal limbs (fracture and contralateral limb) from racing and training fatalities outside this radius are submitted for examination, via overnight courier. Before disarticulation, all limbs are radiographed to confirm the fracture type, with the contralateral limb for comparison. Examination and imaging of the MCPJ occur within 24 h of euthanasia.

**Table 1.** Summary of limb and anatomical site of fracture in the fracture cohort of 18 New Zealand Thoroughbred racehorses.

Bone	Left forelimb	Right forelimb	Total
Humerus	2	0	2
Metacarpal	3	3	6
Proximal phalanx	1	2	3
Proximal sesamoid	6	1	7
Total	12	6	18

### Control cohort

The control cohort consisted of distal limbs from Thoroughbred horses recently (<60 d) in race training that had died because of reasons unrelated to musculoskeletal injury. Nine were euthanized at the completion of racing as they were unsuitable for retraining because of temperament or management issues. Four horses suffered sudden athletic death associated with race training or racing, and the remaining 3 were the result of injury unrelated to exercise.

### Proximal phalanx lesions

**Figure 1.**
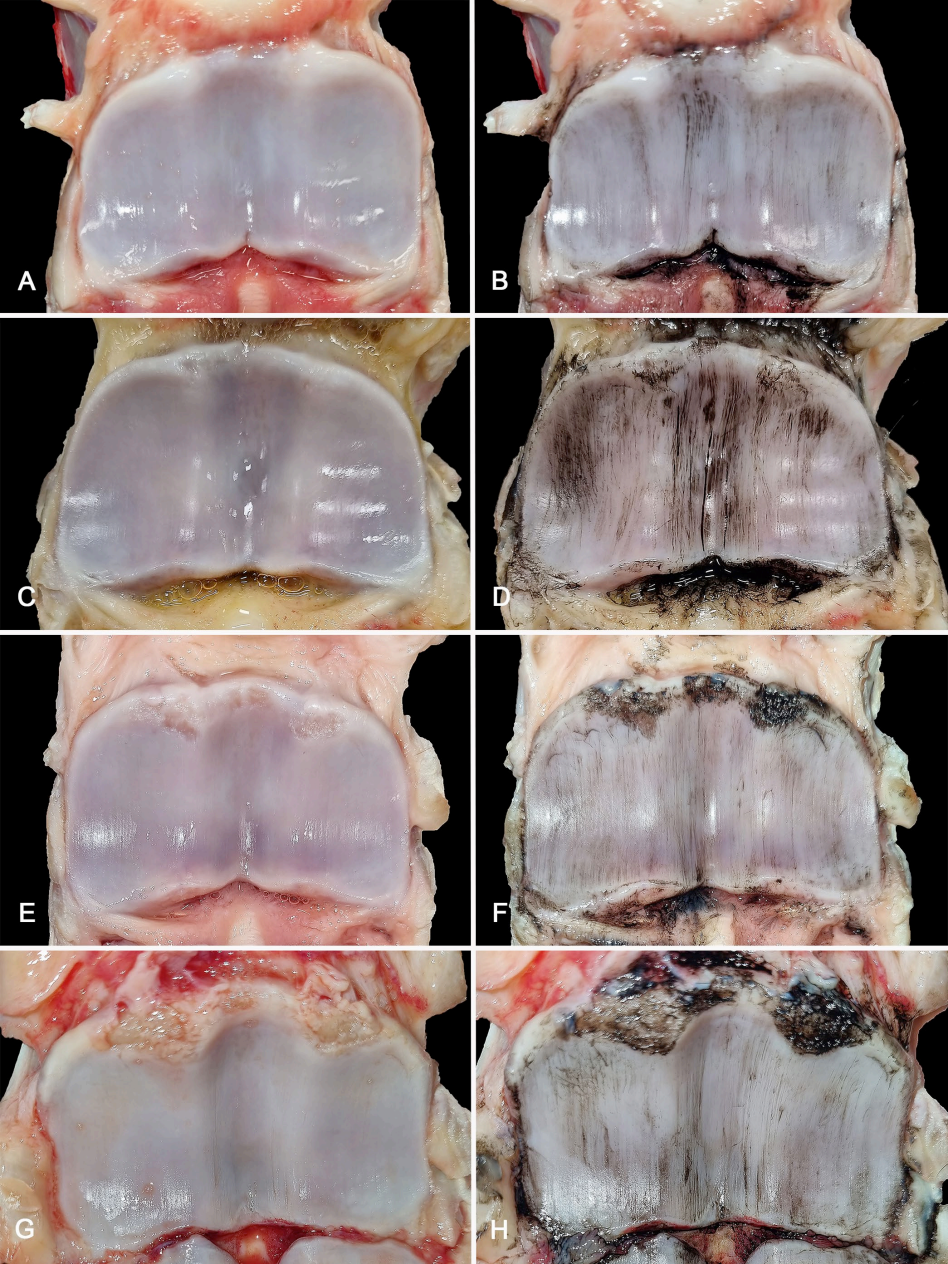
Scoring system for articular cartilage on the dorsoproximal aspect of the proximal phalanx in New Zealand Thoroughbred racehorses. Score 0 = a smooth surface with minimal fibrillation (**A**), and little-to-mild retention of India ink (**B**). Score 1 = small amounts of fibrillation or partial thickness cartilage loss with no subchondral bone exposed (**C**) and mild uptake of India ink (**D**). Score 2 = moderate full-thickness cartilage loss on <70% of the dorsal margin with a focal area of subchondral bone exposure within lesion (**E**) and focal full ink uptake within lesion (**F**). Score 3 = extensive full-thickness cartilage loss distributed across ≥70% of the dorsal margin with associated exposure of the subchondral bone (**G**) and full ink uptake along all the dorsal aspect (**H)**.

**Figure 2.**
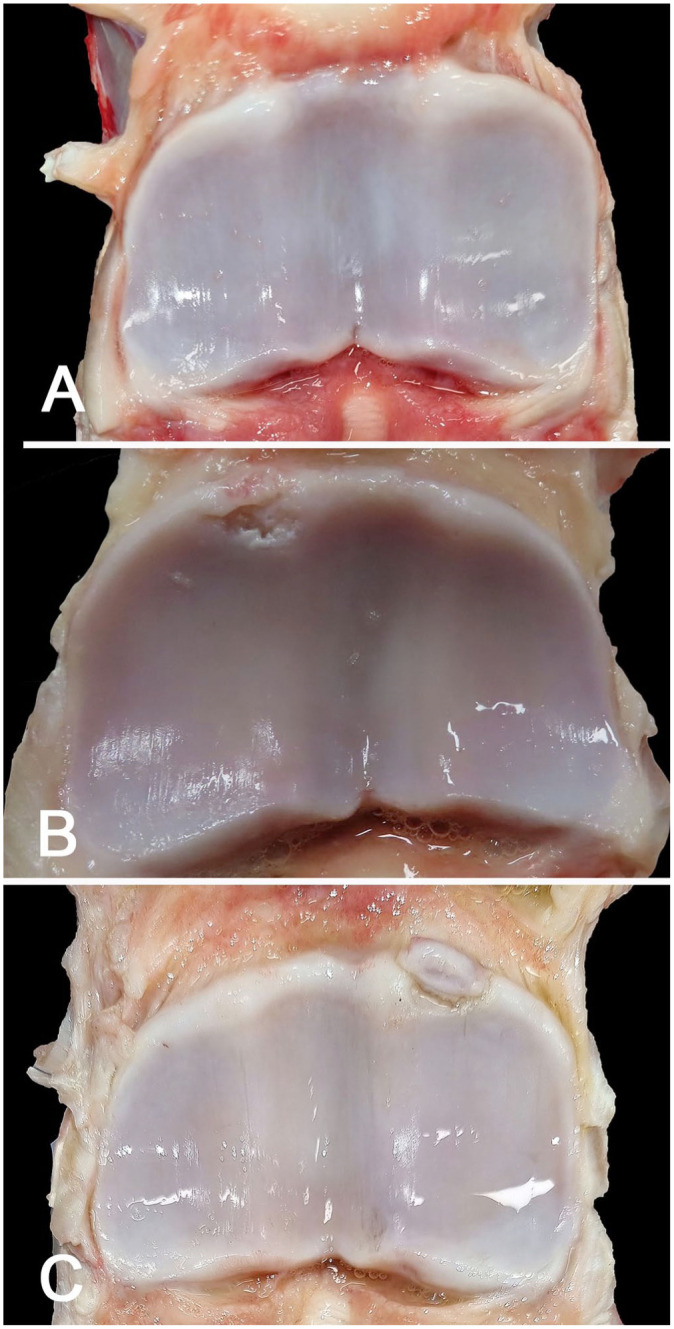
Scoring system for osteochondral chips on the dorsoproximal aspect of the proximal phalanx in New Zealand Thoroughbred racehorses. Scores were defined as (**A**) no osteochondral chip (score 0), (**B**) incomplete osteochondral chip (score 1) to (**C**) complete non-displaced osteochondral chip (score 2).

The MCPJ and MTPJ were resected between the sesamoids and MC3 or metatarsal 3 (**MT3**) from the palmar aspect. The joint surface was wiped with a paper towel to remove synovial fluid and imaged at a focal distance of 30 cm with no zoom (i.e., clean articular surface). The articular surface was then stained with India ink using a paint brush (Black India; Speedball Art Products). After 4 min, excess India ink was wiped from the articular surface with a paper towel following a published protocol^6^ and imaged at a focal distance of 30 cm with no zoom (after India ink application). Articular cartilage was then graded from the images on the medial and lateral aspects using a 3-point scoring system to grade articular cartilage loss (**Fig. 1**). The scoring system was a modification of a system used to score lesions of the distal condyles of MC3 and MT3.^2^ A score of 0 (no abnormalities detected) = the joint was smooth with minimal fibrillation (**Fig. 1A**), and little-to-mild retention of India ink stain on the articular surface with no focal ink staining observed (**Fig. 1B**). A score of 1 (mild) = small amounts of fibrillation or partial thickness cartilage loss on the dorsal aspect with no subchondral bone exposed (**Fig. 1C**) and mild uptake of India ink along the dorsal aspect (**Fig. 1D**). A score of 2 (moderate) = moderate full thickness cartilage loss on <70% of the dorsal margin (ulceration) with a focal area of subchondral bone exposure within the lesion (**Fig. 1E**) and focal full ink uptake within the lesion and mild uptake along most of the dorsal aspect (**Fig. 1F**). A score of 3 (severe) = extensive full thickness cartilage loss distributed across ≥70% of the dorsal margin with associated exposure of the subchondral bone (**Fig. 1G**) and full ink uptake along all of the dorsal aspect (**Fig. 1H**). Osteochondral chip fractures were graded using a 2-point scale from no osteochondral chip (score 0, **Fig. 2A**), incomplete osteochondral chip (score 1, **Fig. 2B**), to complete non-displaced osteochondral chip (score 2, **Fig. 2C**). When we found a fracture in P1, sesamoid, or distal MC3 or MT3, the P1 from the corresponding limb was not graded because of artefact from the fracture. Grading of all specimens was undertaken by an orthopedic equine surgeon (CP Beggan); an equine practitioner who specializes in racehorses, lameness, and poor performance (GO Sommerville); and 2 scientists working in racehorse pathology (MJ Gibson, AS Wehrle-Martinez).

### Data handling

Data describing the horses age, sex, and race starts were obtained from the New Zealand Thoroughbred Racing website (https://nztr.co.nz/) using the horse’s name, brand, or microchip as the identifier.

### Statistical analysis

Statistical analyses were conducted using RStudio (v.4.2.1) with a level of significance set at *p* ≤ 0.05. Descriptive statistics examining the proportion graded at each score were examined between leg, and medial versus lateral aspect using a Fisher exact test. Age and race starts were grouped according to career age as beginning (2–3-y-old), mid (4–5-y-old), or late (5+ y-old). Race starts was grouped based on a median number of 5 race starts for a horse in a season^16^ (0–5, 6–10, and 11+ races). Horse age group, race starts group, and cohort were examined using the Wilcox rank sum test and presented as a median and interquartile range (IQR). One rater was used for the median calculations (GO Sommerville).

Inter-rater reliability of articular cartilage scores and osteochondral chip fractures was assessed using the Gewt coefficient of agreement with ordinal weighting applied (AC2). Intra-rater reliability of articular cartilage scores and osteochondral chip fractures was assessed using the Cohen kappa coefficient. Interpretation of AC2 and Cohen kappa was derived from a proposed system: ≤0.20 = poor, 0.21–0.40 = fair, 0.41–0.60 = moderate, 0.61–0.80 = substantial, and 0.81–1.0 = excellent reliability.^15^

## Results

Statistical differences in age and race starts were not found between horses in the control and fracture cohorts (*p* > 0.05; **Table 2**). Sex, age, and number of race starts of study horses in both cohorts were similar to the base racing population.

**Table 2.** Descriptive data (median and IQR) for 34 Thoroughbred racehorses euthanized because of race-day fractures (fracture) and those euthanized for reasons unrelated to musculoskeletal injury (control).

Variable	Control, *n* = 16	Fracture, *n* = 18	*p*-value	Base racing population
Sex				
Male (gelding, stallion, colt)	7	11		50.5%^10^
Female (filly, mare)	9	7		49.5%^10^
Median age, y (IQR)	5 (4–6)	4 (3–4)	0.187	4 (4–6)^10^
Median race starts, total (IQR)	7 (5–16)	6 (2–12)	0.324	9 (4–19)^16^

Inter- and intra-rater reliability was high for the scores of all 4 articular cartilage variables (**Table 3**). The scores for the medial and lateral osteochondral bone chips had the greatest inter-rater reliability (AC2 > 0.90).

**Table 3.** Gewt coefficient and SE for inter-rater reliability scores and Cohen kappa for intra-rater reliability for cartilage scores in the dorsoproximal aspect of proximal phalanx in Thoroughbred racehorses affected (fracture) and unaffected (control) forelimb fractures.

Variable	Inter-rater agreement	Intra-rater agreement
	Gewt coefficient ± SE	Cohen kappa
Medial cartilage score	0.85 ± 0.02	0.92
Lateral cartilage score	0.89 ± 0.01	0.88
Medial osteochondral chip	0.97 ± 0.01	0.83
Lateral osteochondral chip	0.99 ± 0.004	0.74

Most (82.4%) of the cartilage scores were no abnormalities detected (NAD) or mild (score 1) in all aspects of all limbs (**Table 4**). No significant difference was found in cartilage score among all 4 limbs. Medial versus lateral scores were significantly different in the left forelimbs and right hindlimbs. Osteochondral chips were rarely observed. When present (*n* = 5), osteochondral bone chips were located more commonly on the medial aspect (*n* = 4) rather than the lateral aspect (*n* = 1). One osteochondral chip was the maximum per horse.

**Table 4.** Frequency of cartilage scores by limb in the dorsal aspect of proximal phalanx in 34 Thoroughbred racehorses affected (fracture) and unaffected (control) forelimb fractures.

Score	Left forelimb, *n* = 24 (%)	Right forelimb, *n* = 30 (%)	Left hindlimb, *n* = 24 (%)	Right hindlimb, *n* = 24 (%)	*p*-value between limbs*
Medial cartilage					
0	8 (33)	8 (27)	9 (38)	6 (25)	0.917
1	10 (42)	11 (37)	7 (29)	9 (38)	
2	6 (25)	9 (30)	7 (29)	9 (38)	
3	0 (0)	2 (7)	1 (4)	0 (0)	
Lateral cartilage					
0	15 (63)	13 (43)	18 (75)	12 (50)	0.202
1	9 (37)	15 (50)	6 (25)	12 (50)	
2	0 (0)	1 (3)	0 (0)	0 (0)	
3	0 (0)	1 (3)	0 (0)	0 (0)	
*p*-value (medial vs. lateral within limb†)	0.001	0.130	0.243	0.008	
Medial osteochondral chip					
0	22 (92)	30 (100)	23 (96)	23 (96)	<0.001
1	1 (4)	0 (0)	1 (4)	1 (4)	
2	1 (4)	0 (0)	0 (0)	0 (0)	
Lateral osteochondral chip					
0	35 (100)	30 (100)	23 (96)	24 (100)	<0.001
1	0 (0)	0 (0)	1 (4)	0 (0)	
2	0 (0)	0 (0)	0 (0)	0 (0)	

* *p*-value denotes Fisher exact test for the frequency graded at each score between limbs.

† *p*-value denotes Fisher exact test for the frequency graded at each score within limb (medial vs. lateral).

The articular cartilage score and osteochondral chip fractures were not significantly different between the fracture and control cohorts (*p* > 0.05; **Table 5**). The median articular cartilage score increased with horse age group (medial only) and race start group (Table 5). Horse age group or race start group had no effect on the presence or severity of osteochondral chip fractures (*p* > 0.05). The relationship between horse age and number of race starts was quadratic (*R*^2^ = 0.82).

**Table 5.** Median and IQR dorsal articular cartilage scores of the proximal phalanx by horse age group, race starts group, and cohort of Thoroughbred racehorses affected (fracture, *n* = 18) and not affected (control, *n* = 16) by fatal fracture.

Horse age group	Side	*n*	2–3-y-old	4–5-y-old	6+ y-old	*p-*value, age
No. of horses			12	14	8	
Articular cartilage	Medial	102	0 (0–1)	1 (1–2)	2 (1–2)	<0.001
	Lateral	102	0 (0–0)	0 (0–1)	1 (0–1)	0.152
Osteochondral chip	Medial	102	0 (0–0)	0 (0–0)	0 (0–0)	0.511
	Lateral	102	0 (0–0)	0 (0–0)	0 (0–0)	0.214
Race starts group	Side	*n*	0–5	6–10	10+	*p*-value, no. of races
No. of horses			15	8	11	
Articular cartilage	Medial	102	1 (0–1)	1 (0–2)	2 (1–2)	0.033
	Lateral	102	0 (0–1)	0 (0–1)	1 (0–1)	0.002
Osteochondral chip	Medial	102	0 (0–0)	0 (0–0)	0 (0–0)	0.369
	Lateral	102	0 (0–0)	0 (0–0)	0 (0–0)	0.301
Cohort	Side	*n*	Control	Fracture		*p*-value, cohort
No. of horses			16	18		
Articular cartilage	Medial	102	1 (0–2)	1 (0–2)		0.788
	Lateral	102	0 (0–1)	0 (0–1)		0.707
Osteochondral chip	Medial	102	0 (0–0)	0 (0–0)		0.762
	Lateral	102	0 (0–0)	0 (0–0)		0.393

## Discussion

The horses in our study (both control and fractured) reflect the general New Zealand racing population in age, number of starts, and sex.^10,16^ Therefore, despite the relatively small sample size (~0.8% of the racing population), which is not unusual for a postmortem study, we expect that our results reflect the underlying lesions present in the current New Zealand racing population.

Our scoring system had “excellent reliability” among the 4 raters, according to defined thresholds.^15^ As a result, one rater was used for the median calculations. The rater (GO Sommerville) was selected because he had no input into the generation of the scoring system and is an experienced equine orthopedic surgeon. The higher Gewt coefficient when scoring osteochondral chip fractures reflects the low incidence of chip fractures and highlights the lower ambiguity between criteria for the scores. In contrast, the articular cartilage score may have more subjectivity between raters. The slightly lower Gewt score in the medial versus lateral aspect may be the result of differences in the severity of scores between sides. The medial aspect had a greater number of scores in the 2–3 range, which may suggest that raters were consistently able to detect lesions in the articular cartilage (score > 0), but some variation existed in grading the severity of the erosion.

The greater severity of articular cartilage lesions observed in the medial aspect of both the P1 and MC3 reflects the greater load through the medial aspect of the limb during high-speed exercise.^1,12^ Significant differences in the medial and lateral aspects of P1 were evidenced by the higher cartilage scores on the medial aspect in the left forelimb and right hindlimb, as well as a tendency for higher medial scores in the right forelimb. Similar trends have been reported, with higher lesion grades and areas in the medial versus lateral aspect of P1 when scored for cartilage loss.^6,18^ In addition, the tendency for osteochondral chips in the medial aspect agrees with surgical intervention studies in which most chips are removed from the medial aspect of P1.^32^ Bone lesions on the medial aspect have also been correlated with lesions on the dorsal aspect of MC3 and MT3, further confirming that the mechanism involved is related to the overextension in the MCPJ and MTPJ.^2,7^ Dorsal impact injuries on MC3 and MT3 have been correlated with the presence of POD and follow a trend toward higher scores in the medial condyle.^2^

The significant difference in severity of lesions between the medial and lateral aspects in the left forelimb and right hindlimb follows the pattern of greater loading in these limbs when training in a counterclockwise direction, as is the norm in NZ (74% of race starts). This pattern of training may exacerbate medial-lateral differences in loading compared with the contralateral limbs. Therefore, the lack of difference in cartilage scores between left and right limbs was surprising, given the medial versus lateral differences in the left forelimb and the tendency for P1 fractures to occur in the left forelimb when racing counterclockwise.^11,21^ However, the lack of difference in scores between left and right limbs within medial and lateral lesions is in agreement with studies assessing POD scores in UK racehorses and articular cartilage in P1s of the Kaimanawa feral horse population.^2,6^ Most fractures in P1 originate in the parasagittal groove; articular cartilage lesions are less frequent in the groove, but the load is greater in the groove compared with the medial and lateral aspects of P1.^7^ Given the lack of difference in lesions between horses in the fracture and control cohorts, the grade of articular cartilage may not be an indication of fracture risk. Differences in lesion severity may exist between the affected and contralateral limb; however, P1 may not have been scored when the fracture site was in the MCPJ because of the artifacts caused by bone fragments.

Well-established risk factors for catastrophic limb fracture are older age and a higher number of race starts.^11,13,29^ A longer racing career results in more high-speed load cycles and an accumulation of microdamage, which increases fracture risk.^17^ We found that increases in age and race starts were associated with a greater severity of cartilage damage. Similarly, studies of MCPJ and MTPJ lesions in UK racehorses found that more race starts and greater age were associated with an increase in POD score.^3,18^ In the feral Kaimanawa horse population, the presence of osteoarthritis in the MCPJ has also been observed with increasing age.^6^ Therefore, the increase in cartilage score may not only be from an accumulation of race starts, but reduced ability for articular cartilage regeneration as the horse ages.

Our cartilage scoring system had excellent inter-rater reliability. Overall, the lesion graded was mild with few high scores allocated. The tendency for a higher score in the medial aspect and increasing score with age and race starts reflects the greater load through the medial aspect and reduced capacity of articular cartilage to repair as the horse ages. We found no association between articular cartilage score and fracture despite the lesions reported reflecting focal sites of load and response within P1. Lack of detailed clinical history before euthanasia restricted our ability to assess an association between cartilage score and impaired performance or function. Application of this scoring system to other larger datasets would permit greater statistical power to correlate P1 articular cartilage lesions with age, race starts, and clinical history.
